# Progress in the study of oxidative stress damage in patients with schizophrenia: challenges and opportunities

**DOI:** 10.3389/fpsyt.2025.1505397

**Published:** 2025-02-21

**Authors:** Kaiguo Zhang, Lijing Zou, Yi Cai

**Affiliations:** Shenzhen Mental Health Centre, Shenzhen Kangning Hospital, Shenzhen, China

**Keywords:** schizophrenia, oxidation and antioxidant, oxidative stress, treatment, prognosis

## Abstract

Schizophrenia is a serious psychiatric disorder of multifactorial triggers, with a lifetime prevalence of about 1% in all countries of the world, with a slightly higher prevalence in males than in females, and with a peak incidence between the ages of 15-35 years old, and a poor prognosis for most of the patients, which imposes a heavy burden on the society and the family. Early intervention is important for the prognosis and regression of patients. More and more studies have found that the imbalance of oxidation and antioxidant, and the persistent damage to the brain by oxidative stress play an important role in the occurrence and development of schizophrenia. Antioxidants, as additive therapy, play an important role in improving symptoms as well as preventing relapse in patients with schizophrenia. This paper intends to address the pathogenesis of oxidative stress injury and schizophrenia, and the significance of oxidative stress in the treatment of schizophrenia.

## Introduction

Schizophrenia is a chronic, severe, and psychiatric disorder of multifactorial onset that affects approximately 1% of the world’s population (1). It is characterized by positive symptoms (e.g., hallucinations and delusions), negative symptoms (e.g., depressed mood, laziness and social withdrawal) and cognitive impairment (2). The course of the disease is often prolonged, accounting for more than half of psychiatric inpatients, and about half of the patients eventually develop psychiatric disability, which places a heavy burden on society and families. Although most patients are diagnosed in adulthood, cognitive impairment and social deficits may appear as early as adolescence (3, 4). A growing number of studies have found that oxidative stress damage, which occurs since early childhood, sustains damage to the brain, affects neuronal growth and development, and influences neuronal structure and function, and may play an important role in the pathophysiological mechanisms of schizophrenia (5–7).

## Oxidation and antioxidant

**Figure 1:**
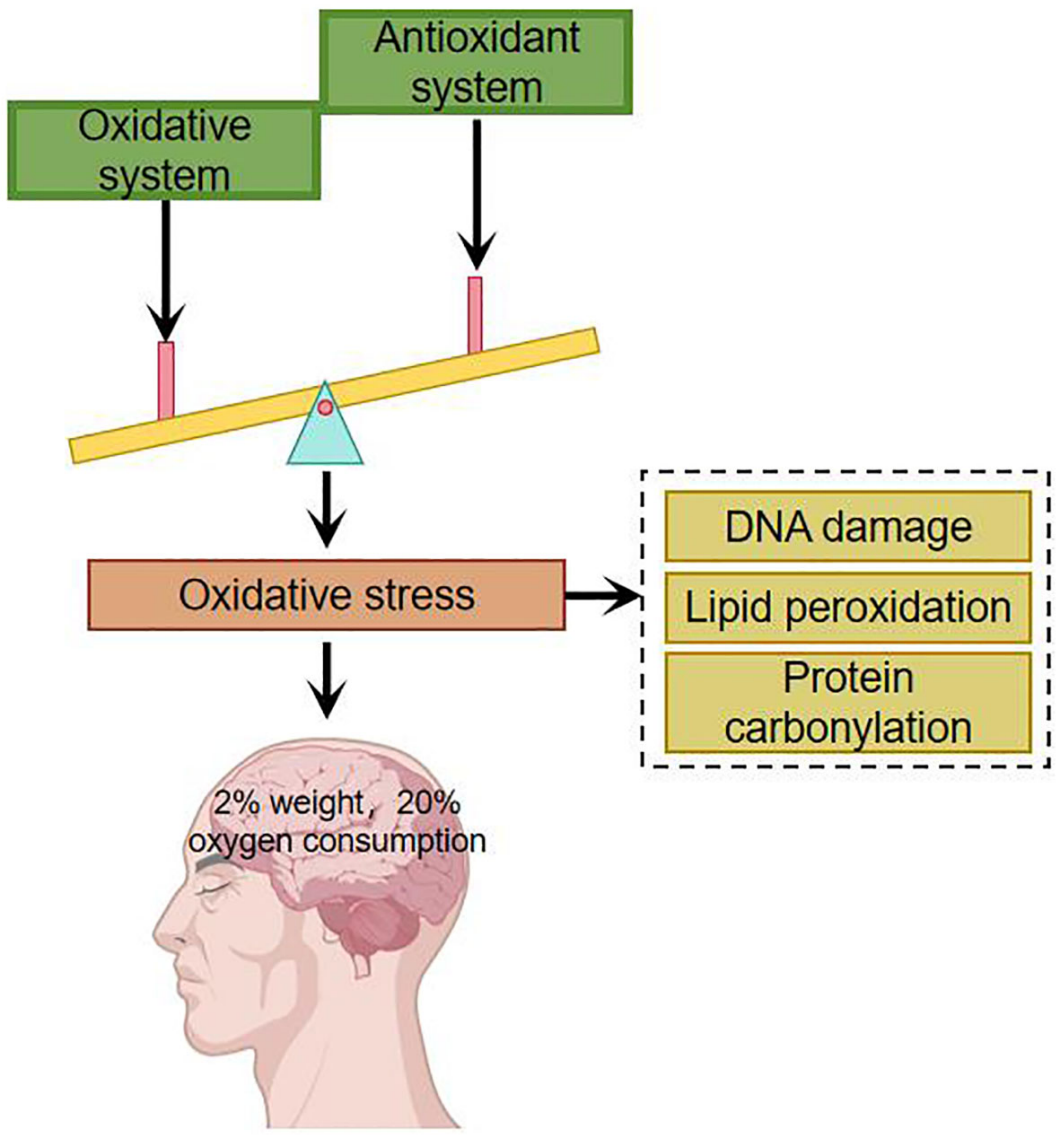
Imbalances in the oxidative and antioxidant systems have a particularly significant impact on the brain.

Under normal conditions, there is an oxidative system and an antioxidant defense system in the human body, both of which are in dynamic equilibrium. The oxidative system includes: superoxide anion, hydroxyl radicals, etc. The antioxidant defense system includes: superoxide dismutase, catalase and other enzymatic antioxidants as well as non-enzymatic antioxidants such as albumin, uric acid and glutathione. Oxidative stress occurs when the organism is subjected to a variety of internal and external environmental stimuli and there is an imbalance between oxidative and antioxidant processes. Appropriate oxidative stress is necessary for the normal physiological function of the organism, and excessive oxidative stress may have deleterious effects. The main targets of oxidative stress damage are DNA, lipids and proteins (8–10). Oxidative stress damage can lead to mutations in DNA by inducing nucleic acid strand breaks and purine oxidation, damage to cell membranes and organelle membranes through lipid peroxidation, and it can also lead to denaturation of proteins, which loses its original function and affects the structure and function of cells (11). The brain accounts for 2% of the body’s total weight but consumes about 20% of the body’s oxygen (**Figure 1**). Compared with other organs in the body, the brain consumes more oxygen, generates more free radicals, has a higher concentration of polyunsaturated fatty acids, and a lower level of antioxidants, and is therefore more susceptible to damage caused by oxidative stress (12).

## Oxidative stress damage may be linked to schizophrenia mechanisms

**Figure 2:**
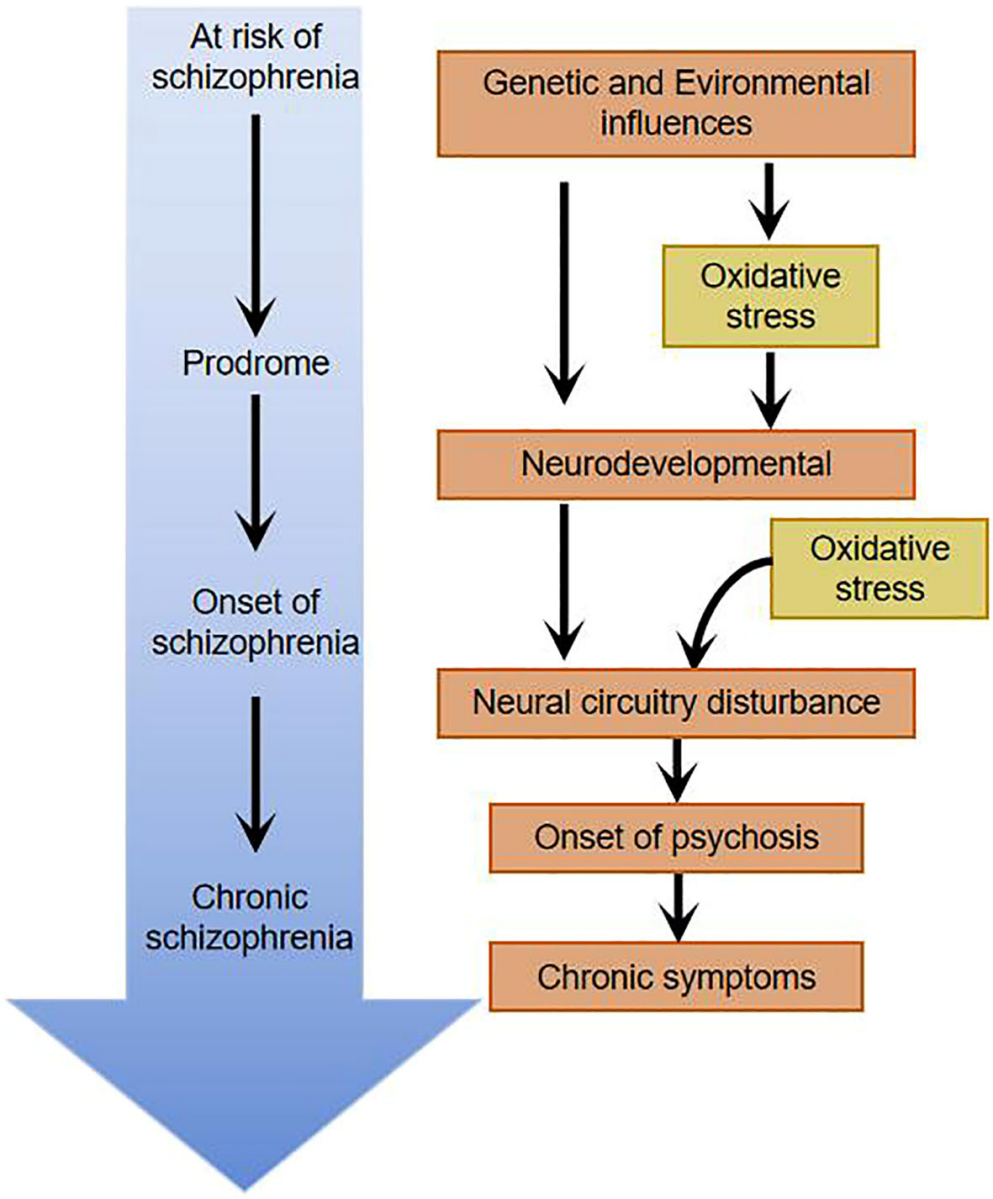
Oxidative stress damage throughout the course of schizophrenia.

Social isolation, vitamin D deficiency, chronic stress during adolescence, perinatal infections, inflammation, etc.-all of which ultimately lead to oxidative stress (5, 13). Under normal conditions, the body’s oxidation and antioxidant activity are in dynamic equilibrium; under abnormal conditions, free radicals that are not scavenged from the body can react with normal tissue cells, damaging the cellular structure and causing cellular dysfunction. Measurement of the concentration of free radicals in the body is impractical because of their chemically reactive nature resulting in low levels and short half-lives in the body. In clinical studies, the level of oxidative stress is reflected indirectly by antioxidant enzymes, non-enzymatic antioxidants, and peroxidation products. 1954 Hoffer et al. suggested for the first time that oxidative stress damage plays an important role in the pathogenesis of schizophrenia by examining the levels of oxidative stressors in patients. Studies of oxidative stress damage in schizophrenic patients have been conducted mainly through blood (e.g., leukocytes, lymphocytes, albumin, uric acid, etc.), cerebrospinal fluid, and postmortem brain tissue of patients. Researchers have found that schizophrenic patients have higher peroxidation products detectable in peripheral blood and cerebrospinal fluid than the normal population, suggesting that schizophrenic patients have higher oxidative stress damage in their organisms than the normal population (14, 15). In order to exclude the influence of medication and disease duration on the study, some researchers studied patients with first-episode unmedicated schizophrenia and found that markers of oxidative stress damage were significantly elevated compared to the normal population (16) and that there was a significant correlation between the severity of psychiatric symptoms and markers of oxidative stress damage (5, 17). Researchers have found that serum superoxide dismutase (SOD) is significantly lower and catalase (CAT) and malondialdehyde (MDA) levels are significantly higher in patients with schizophrenia compared to the normal population (18). Studies of brain tissue from postmortem patients have clearly identified oxidative stress damage (19, 20), but confounding factors, such as the effect of other somatic diseases comorbid before death, cannot be excluded. Schizophrenia is a disorder that mostly develops in young adulthood, and testing the brains of elderly patients after death does not truly reflect the pathophysiological changes in the patients’ brains. A major advancement in research in recent years has been the use of MRS to study brain tissue from living subjects, where researchers have found that glutathione (GSH) levels in the medial prefrontal cortex of patients with first-episode schizophrenia who were not on medication were reduced by approximately 52% compared to the normal population (20). Do KQ et al. studied cerebrospinal fluid from first-episode unmedicated patients and found that GSH levels were reduced by 27% compared to the normal population (21), and Yao et al. measured the caudate nucleus of postmortem patients and found a 41% reduction in GSH levels (22). The brain, as 20% of the oxygen consumption in the organism, undergoes redox reactions all the time, generating a large number of free radicals. The active free radicals in the brain produce neurotoxic effects and sustain damage to nerve cells, causing peroxidation of cell membranes and organelle membranes, impaired membrane fluidity and stability, affecting nerve cell plasticity and neurotransmitter vesicle transmission, and affecting chemical reactions taking place in nerve cells, which may accelerate or lead to schizophrenia and a range of clinical manifestations (**Figure 2**).

The classical dopamine hypothesis of schizophrenia suggests that schizophrenics have central dopamine hyperfunction compared to normal individuals. Whereas the metabolism of dopamine generates large amounts of hydrogen peroxide, hydrogen peroxide generates reactive oxygen radicals via auto-oxidation of dopamine. These reactive oxygen species may subsequently interact with superoxide dismutase and glutathione, leading to reduced levels of these antioxidants, making the brain’s ability to cope with oxidative stress damage much reduced and further accelerating neuronal cell damage in the corresponding brain regions (23). Glutathione (GSH) redox system plays an important role in reducing the damage caused by oxidative stress in the organism. GSH is one of the major free radical scavengers in the brain, which can be converted to oxidized glutathione (GSSG) by glutathione peroxidase (GPx) to deplete reactive oxygen radicals, and GSSG can then be reduced to GSH by glutathione reductase (GR).Therefore, measurement of the body GSH, GSSG and their related enzyme responses are important for assessing oxidative stress injury. A study comparing GSH, GSSG, GPx and GR levels in the caudate region of the brain after death in schizophrenia patients and normal subjects showed that the schizophrenia group had significantly lower levels of GSH, GPx, and GR than the control group, which did not have any psychiatric disorders (24).The potential mechanism by which the reduced level of GSH affects the morphology of neurons may be through oxidative stress damage inducing lipid peroxidation, the protein denaturation, which further leads to cell membrane alterations and cytoskeletal changes, affecting the normal functioning of neuronal cells in the corresponding brain regions involved in schizophrenia (25).

The glutamate hypothesis of schizophrenia suggests that the function of N-methyl-D-aspartate (NMDA) receptors is impaired in this disorder. This hypothesis derives from the fact that drugs, including phenylcyclohexylpiperidine (PCP), cause schizophrenia-like psychotic symptoms in humans by blocking the function of the NMDA receptor, and that such psychotic symptoms are difficult to diagnostically distinguish from schizophrenia (26). Glutamate, as the main excitatory neurotransmitter in the central nervous system, also has a high level of excitotoxicity, and this excitotoxicity is mainly affected through oxidative stress damage, and its toxic effects on cells are mainly through two mechanisms: one is that the influx of calcium ions may trigger the infiltration of isotonic fluids, which can subject the cells to mechanical stress, which can in turn affect the function of the cells or even lead to cell death. The other is that the excitatory cycle induced by the entry of large amounts of calcium ions into the cell stimulates the further release of glutamate, the latter of which may lead to the further exacerbation of oxidative stress injury (27).

**Figure 3:**
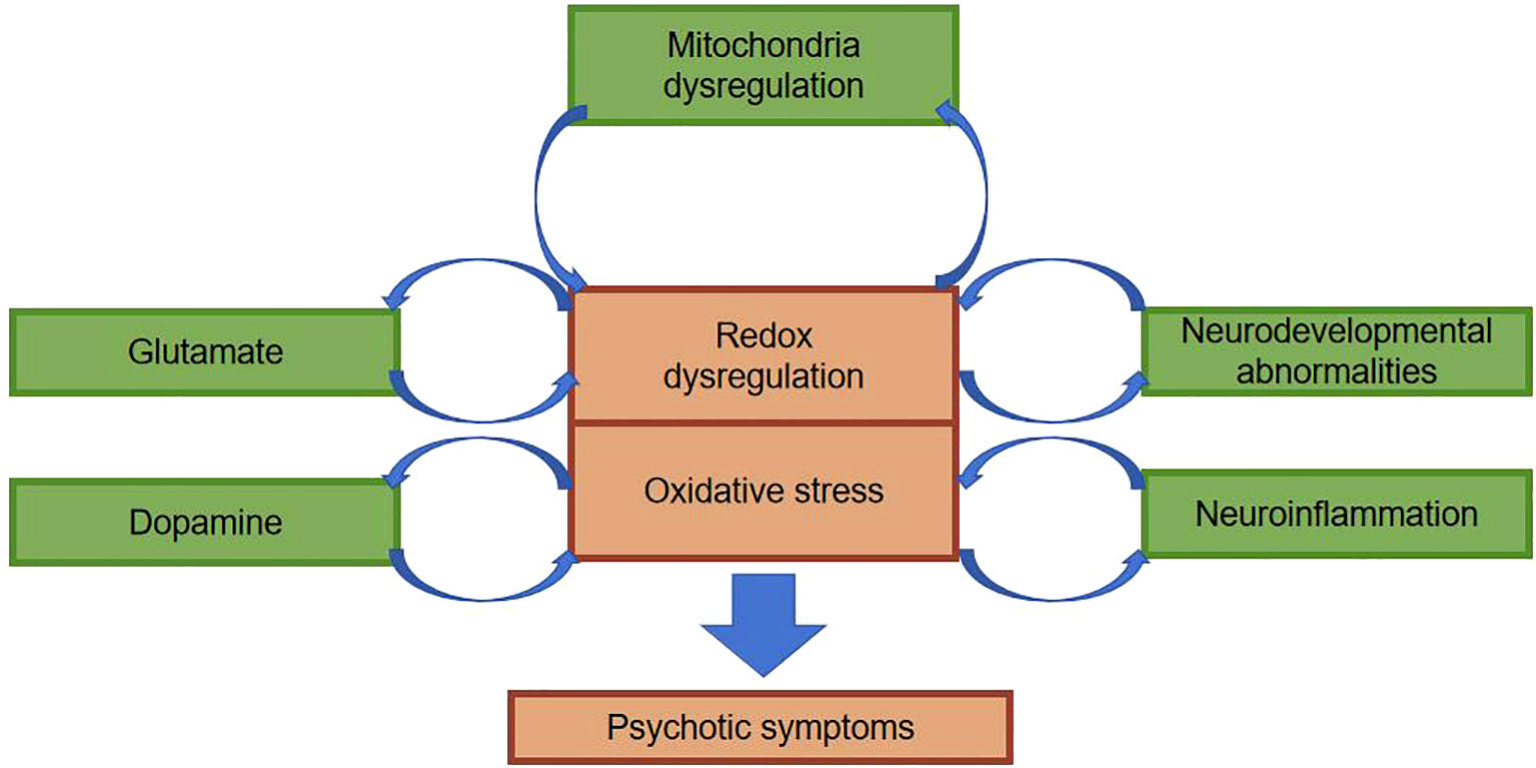
Possible mechanisms by which oxidative stress affects schizophrenia.

Previous studies on schizophrenia has predominantly concentrated on dopaminergic and glutamatergic neurotransmitters. Recently, the neuroinflammatory theory puts forth a viewpoint that dysfunction of the body’s immune system as a consequence of genetic and environmental factors may be a paramount pathologic mechanism in schizophrenia (28, 29). Interactions between neuroinflammation and oxidative stress affect the functional integrity of a neuron as well as the integrity of the meshwork surrounding that neuron. Aside from that, the association between chronic inflammation and oxidative stress facilitates the release of pro-inflammatory cytokines, the stimulation of astrocyte activity, as well as the dysregulation of dopaminergic and glutamatergic pathways, which ultimately gives rise to the onset of schizophrenic symptoms (**Figure 3**) (30). As already demonstrated by relevant imaging studies, during schizophrenic episodes, changes occur in brain structure, most commonly manifested as a reduction in hippocampal and cortical volume, accompanied by ventricular enlargement, which is independent of pharmacological treatment (31). In contrast to Alzheimer’s disease, the schizophrenia brain changes are not progressive neuronal degeneration and death, but rather changes in brain organization and in the size of neurons and other brain cells. Molecular studies have displayed altered expression of inflammatory markers in the prefrontal and temporal cortex and hippocampus of schizophrenic patients. This phenomenon suggests that the body may have oxidative stress as well as altered inflammatory responses. Clozapine, featured by its enduring immunosuppressive effects and anti-inflammatory properties that alleviate microglial activation, is associated with its adverse effect of agranulocytosis, which is also correlated with its anti-inflammatory action and abatement of oxidative stress damage. As a consequence, lessening inflammatory response and oxidative stress damage is expected to be a target goal for the future treatment of schizophrenia (32).

The central nervous system contains about 100 billion neurons. Astrocytes, oligodendrocytes, and microglia, as well as other cell types, collectively make up the adult brain and spinal cord. Oxidative stress damage early in life may disrupt the normal developmental stages of the brain (**Figure 3**). In particular, synapses form and connect, and these changes ultimately result in abnormal information processing and, ultimately, to schizophrenia (33). It’s particularly noteworthy that there are dramatic abnormalities in brain development and maturation in patients with schizophrenia spectrum disorders. First and foremost, a multitude of neuroblasts fail to reach the correct location during the 4-6 months of gestation, and autopsy studies reveal that they are buried deep in the white matter of the brain (34). On top of that, during childhood and adolescence, individuals in the pre-schizophrenic phase show excessive loss of neuronal and synaptic connectivity to the extent that, during psychotic episodes, about one-third to one-half of patients show marked atrophic changes and enlargement of the lateral ventricles (33, 35). The enlarged ventricles mirror the loss of brain tissue in schizophrenic patients: oxidative stress damage continues after psychotic episodes, the loss of brain volume continues to worsen, and appears to correlate with the duration of untreated psychosis and the number of exacerbations. Patients suffering from schizophrenia are in an exacerbation phase, where oxidative stress damage persists and keeps damaging the patient’s brain, which exerts a paramount impact on the patient’s prognosis. In addition, some scholars have reported that the patient’s prognosis worsens the longer the condition stays untreated (36, 37).

It has been found that micronutrient imbalances are present in patients with schizophrenia (38). Iron ions play an important role in the body, with nearly two-thirds of its total amount present in hemoglobin. Most of the remaining iron ions are located in the brain, liver, spleen and heart. Iron ions are adept at catalyzing redox reactions. Transferrin and ferritin are iron transport proteins, both with antioxidant properties. These two proteins act as antioxidants and reduce oxidative stress damage by lowering the concentration of free iron ions. It has been found that serum levels of iron, ferritin and transferrin are significantly lower in schizophrenic patients compared to normal controls, which also suggests that oxidative stress damage may play an important role in the development and progression of schizophrenic patients (39).

Impaired cognitive function is one of the important symptom clusters of schizophrenia, which mainly manifests impaired attention, memory, visual and verbal learning ability, etc. More and more studies have found that oxidative stress injury is involved in the pathological process of cognitive dysfunction in schizophrenia patients (16, 40). It has been found that the higher the severity of cognitive impairment in patients with schizophrenia, the lower the body’s antioxidant capacity, the increased protein oxidation in the brain, and the more severe the cognitive impairment (41, 42). Some researchers have studied first-degree relatives of schizophrenic patients and similarly found impaired cognitive functioning compared to the normal population (43). It is suggested that in clinical practice, reducing the level of oxidative stress in patients with early-onset schizophrenia may be beneficial in improving their cognitive function (44, 45).

## Oxidative stress and schizophrenia treatment

Oxidative stress is amenable to early intervention and treatment, and there is a large therapeutic potential in this area. The effect of antipsychotic drugs on oxidative stress in the body is not yet clear, and more studies suggest that antipsychotic drugs play an important role in reducing oxidative stress damage in the body (46, 47). A prospective study conducted by Meng-Chang et al. at the Department of Psychiatry, Chang Gung Hospital, Taiwan, found a significant negative correlation between the scores of positive and negative symptom scales in the acute phase of schizophrenia and the level of antioxidants in the organism, and after a 4-week antipsychotic medication, the level of oxidative stress in the organism was significantly reduced compared to the previous one, suggesting that antipsychotic medication may have an effect on the organism’s oxidative stress levels (48). A study conducted by Cristiano et al. involving more than 100 patients treated with antipsychotic medication for 11 weeks found that risperidone was able to significantly reduce oxidative stress levels, and that there was no correlation between the decrease in oxidative stress levels and symptomatic improvement, whereas there was a correlation with the use of risperidone, which ruled out an effect of psychiatric symptomatic improvement on oxidative stress levels (49). oxidative stress levels (40).Parikh et al. found increased levels of lipid peroxidation in the brains of rats chronically treated with haloperidol, whereas no such changes were detected in animals chronically treated with risperidone (as well as olanzapine and clozapine) after 90 days, suggesting that the effects of different antipsychotic medications on oxidative stress may also vary (50). 2022 Goh et al. conducted a Meta-analysis study that included 71 papers involving 4588 patients with schizophrenia and 3983 healthy controls, and found that schizophrenia is associated with an impaired organismal antioxidant defense system, especially with regard to non-enzymatic antioxidants. In addition, antipsychotic drugs and their discontinuation may also alter enzymatic and non-enzymatic antioxidant defense systems. Different types of samples (erythrocytes, plasma, serum, and whole blood) and different measurement techniques may lead to differences in the results of antioxidant activity assays, and confounding factors such as the patient’s age, gender, disease duration, comorbidities with other diseases, and patient condition may have an effect on antioxidant activity (6, 51, 52).

Antioxidants are able to play a role in the development, progression and treatment of schizophrenia by scavenging reactive oxygen radicals and reducing the level of oxidative stress in the body. Antioxidants such as vitamin E, N-acetylcysteine (NAC), and melatonin have been investigated as adjunctive treatments for schizophrenia, where they can reduce oxidative stress damage and improve patients’ symptoms (53). In a previous study of an animal model of schizophrenia (Gclm knockout mice), NAC was given to counteract oxidative stress damage in the early stages of the disease, and the abnormal behavior of the animal model improved (54). A review of the literature on NAC for the treatment of schizophrenia has revealed that NAC is an effective adjunct to the treatment of schizophrenia, improving psychotic symptoms and cognitive functioning, and additionally this therapeutic effect appears to play a role in schizophrenia at all stages of the disease (55, 56). Studies on hesperidin, a naturally occurring antioxidant and neuroprotective flavonoid, have revealed that hesperidin reduces the level of oxidative stress in prefrontal cortex, striatum and hippocampal regions, and prevents and reverses ketamine-induced schizophrenia-like behavior in mice. It is suggested that hesperidin can be used as a nutritional supplement to prevent as well as assist in the treatment of disorders such as schizophrenia as an important agent in nutritional psychiatry (57). It has been found that appropriate supplementation of the diet with foods containing antioxidants can be helpful in the treatment of schizophrenic patients, and omega-3 polyunsaturated fatty acids, among others, are often used as antioxidant potentiators in the treatment of schizophrenia, which have been found to prevent neuronal cells from suffering from oxidative stress damage, mainly by enhancing the stability of lipid membranes (58–60). Similarly, combination therapy with ginkgo biloba extract and haloperidol has been reported to achieve better efficacy, enhancing the effectiveness of antipsychotics and reducing some extrapyramidal side effects (44). Digoxin, commonly isolated from various plants, is a citrus nutrient that has been shown to increase intracellular antioxidant capacity and alleviate symptoms associated with neurological disorders. One researcher found that digoxin applied to mice prevented schizophrenia-like behaviors and attenuated oxidative stress in ketamine schizophrenic mice (61). Oxidative stress is more prominent in the early stages of schizophrenia disease, and researchers hope to use oxidative stress indicators as an important reference for measuring the severity of the disease in the early stages as well as the efficacy of subsequent treatment (62). Advances in neuroimaging techniques, particularly the measurement of glutathione (GSH) levels in the brain, which are important for antioxidant defense, by magnetic resonance, have gradually made this idea a possibility. A growing number of studies have found that reducing the body’s level of oxidative stress, intervening early in the treatment of people at risk for schizophrenia, and reducing the duration of the untreated period can slow down the deterioration of cognitive functioning, improve psychotic symptoms, enhance patients’ recovery, and may even change the trajectory of the disease. Early cognitive-behavioral therapy can be administered to adjust the patient’s lifestyle, and if necessary, small-dose antipsychotic medication can be given to reduce the level of oxidative stress in combination with antioxidants and other agents to slow down neuronal cell damage.

## Summary

The etiology of schizophrenia has not yet been clarified, and more and more studies have shown that schizophrenia is a progressive neurodevelopmental disorder, and multiple factors play a role in the onset and development of the disease. The level of oxidative stress in the brain of schizophrenic patients is significantly higher than that of the normal population, and the level of enzyme and non-enzymatic antioxidants is significantly decreased, especially in the acute phase, when the symptoms of hallucinatory delusions are obvious, and the organism’s oxidative stress damage is more severe relative to the smooth phase. However, the exact molecular mechanisms of the disease are unknown. The imbalance between oxidative and antioxidant causes the internal environment in which neurons live to be compromised, affecting substances such as lipids, proteins and nucleic acids in neuronal cells, and affecting neuronal structure as well as function. Early intervention therapy with antioxidant supplementation to reduce the level of oxidative stress and oxidative stress damage may provide a new breakthrough point in the treatment of schizophrenia.
